# Brain-Derived Neurotrophic Factor Val66Met Polymorphism Is Associated With a Reduced ERP Component Indexing Emotional Recollection

**DOI:** 10.3389/fpsyg.2019.01922

**Published:** 2019-08-21

**Authors:** Rhiannon Jones, Gavin Craig, Joydeep Bhattacharya

**Affiliations:** ^1^Department of Psychology, University of Winchester, Winchester, United Kingdom; ^2^Department of Psychology, University of Westminster, London, United Kingdom; ^3^Social, Genetic & Developmental Psychiatry Centre, Institute of Psychiatry, Psychology & Neuroscience, King’s College London, London, United Kingdom; ^4^Department of Psychology, Goldsmiths, University of London, London, United Kingdom

**Keywords:** BDNF Val66Met, genetic, emotion, memory bias, recollection, old/new, LPC

## Abstract

The Met allele of the brain-derived neurotrophic factor (BDNF) Val66Met polymorphism is associated with reduced functioning of the amygdala and hippocampus. It has been linked to major psychiatric conditions, including depression and post-traumatic stress disorder, and is associated with deficits in episodic memory. The precise mechanisms of the BDNF gene’s influence on emotional memory are not well characterized, especially its impact on recognition. Two electrophysiological experiments of emotional memory were run on two independent samples genotyped for BDNF Val66Met. Event-related potentials (ERPs) corresponding to the recognition of negative and neutral words (Experiment 1, *N* = 37) and negative and positive words (Experiment 2, *N* = 23) were recorded, and the late parietal component (LPC), typically associated with conscious recollection, was analyzed. In Experiment 1, a reduced LPC was observed in Met carriers (*N* = 12) compared to Val homozygotes *(N* = 25) in the negative condition, but the group difference was not present in the neutral condition. In Experiment 2, the reduced LPC was seen in Met carriers (*N* = 12) compared to Val homozygotes (*N* = 11) across both conditions. This study provides the first evidence of an association between the BDNF Val66Met genotype and the late parietal electrophysiological component, suggesting that the conscious experience of emotional recollection may differ according to BDNF Val66Met genotype. Further, these results suggest that this effect is likely due to emotional arousal rather than valence polarity. Results were discussed with reference to the possible mechanisms by which emotional recollection deficits may contribute to psychopathology.

## Introduction

A commonly found single nucleotide polymorphism (SNP) of the brain-derived neurotrophic factor (BDNF) gene has been strongly linked to both episodic memory and psychopathology. This valine (Val) to methionine (Met) amino acid substitution at codon 66 (Val66Met) is associated with reduced hippocampal volume (e.g., Egan et al., 2003; Schofield et al., 2009), and behavioral and neural differences during memory tasks have been consistently reported, with Met carriers showing reduced performance, often accompanied by reduced hippocampal activity (Egan et al., 2003; Hariri et al., 2003; Cathomas et al., 2010; Kambeitz et al., 2012). Moreover, evidence suggests that this polymorphism has a particular impact on emotional episodic memory (Cathomas et al., 2010; Molendijk et al., 2012).

Brain-derived neurotrophic factor Val66Met has been linked to a variety of psychopathologies, including major depressive disorder (Legge et al., 2015), bipolar disorder (Cao et al., 2016) schizophrenia (Notaras et al., 2015), and post traumatic stress disorder (Felmingham et al., 2013). The Met allele is often linked to cognitive or neural differences within patient samples (Cao et al., 2016), or diminished therapeutic response (Felmingham et al., 2013; Notaras et al., 2015), suggesting that its role in psychopathology may be an indirect consequence of its role in cognitive functions such as memory and emotional processing.

While there is a wealth of fMRI research into the influence of BDNF Val66Met on episodic memory, the slow response of hemodynamic methods precludes examination of its complex and dynamic constituents. Within recognition memory, rapid but functionally separable processes have been identified using electroencephalographic (EEG) methods (Rugg and Curran, 2007), which can be selectively affected by genetic variants (Ross et al., 2015) and associated with particular memory biases (Danion et al., 2005; Neumann et al., 2007). Therefore, the primary objective of our study was to characterize the temporal dynamics of genotype effects on emotional recognition. Dual-process theories (e.g., Yonelinas, 2001) of recognition proposes the functional separation of familiarity – a sense of recognition in the absence of specific memories of the learning episode, and recollection – re-experiencing the initial event with vivid reinstatement of detail and contextual features. Evidence from event-related potentials (ERPs) have linked these processes to specific ERP components: (i) the FN400, in which successfully remembered items elicit a less negative wave than new items over mid-frontal areas 300–500 ms post-stimulus, is associated with familiarity (Rugg and Curran, 2007; although see Paller et al., 2007); (ii) the late parietal component (LPC), in which successfully remembered items elicit a more positive-going wave than new items over left-lateralized parietal areas 500–800 ms post-stimulus is associated with recollection (Woodruff et al., 2006; Rugg and Curran, 2007; Yu and Rugg, 2010). As evidence from combined ERP/fMRI (Hoppstädter et al., 2015) and patients with hippocampal lesions (Düzel et al., 2001; Addante et al., 2012) support the role of hippocampal networks in generation of the LPC but not the FN400, we strategically focused our analysis on the LPC amplitude.

The enhancement of memory for emotional events is well documented (e.g., Bradley and Lang, 1994), and memory of emotional compared to neutral stimuli tends to be more vivid (Todd et al., 2012) and accurate (Labar and Cabeza, 2006). The two primary explanations for this enhancement are the modulation model (McGaugh, 2004; Schmidt and Saari, 2007), and mediation theory (Talmi et al., 2007), which work in a complementary fashion to explain long term (hours) and shorter term (minutes) enhancement, respectively. The modulation model of emotional memory (McGaugh, 2004; Schmidt and Saari, 2007) states that enhanced consolidation occurs due to the activation of the amygdala following adrenaline and cortisol secretion during emotional arousal. However, as the enhanced memory for emotional material occurs within minutes rather than hours, and is observed for emotional stimuli compared to neutral even when they are encountered in the same encoding context, this model provides only a partial explanation of emotional memory enhancement (see Talmi, 2013). Mediation theory (Talmi et al., 2007) suggests that while long-term enhancement of emotional memory can be explained by the modulation model, early enhancement is due to the preferential recruitment of cognitive resources which leads to a deeper processing of emotional material; for example, emotional material receives more attention, is more distinctive, and is more likely to be automatically evaluated in terms of thematic links (see Talmi, 2013). On a neural level, this preferential recruitment is suggested to be triggered by the amygdala (LeDoux, 2000), and leads to an increased activation of the medial temporal lobes. This explains findings of amygdala activation, and the correlation between amygdala and temporal lobe activation (Dolcos et al., 2012), predicting subsequent memory of emotional memories; also the impairment of emotional memory due to amygdalar lesions (Labar and Cabeza, 2006).

Although both of these models focus on the neural dynamics during encoding, recent evidence of a decoupling between attention at encoding and subsequent memory suggests that retrieval may have a more active role in emotional enhancement than previously thought (Shafer and Dolcos, 2014; Barnacle et al., 2018). The emotional context maintenance model (Talmi et al., 2017), for example, suggests that when an emotional item is retrieved at test, the retrieval of the emotional context makes it more likely that further emotional items will be retrieved due to their contextual similarity. Emotionally enhanced memory is associated with increased activation of amygdala and hippocampal regions not only during encoding but also during retrieval (see Dolcos et al., 2012), and evidence from Shafer and Dolcos (2014) shows that this is not only due to incidental encoding which happens at the time of retrieval, but is also instrumental in the retrieval success.

The emotional memory literature, therefore, highlights the crucial role of the amygdala in the enhancement of memory for emotional stimuli, which works with the hippocampus as well as networks underlying attentional or contextual processing, at both encoding and retrieval phases. While the effect of the BDNF Val66Met genotype on the amygdala and emotion processing has not been as widely researched as its effect on non-emotional memory and the hippocampus, some studies do point to a modulation. For example, smaller amygdala volume (along with smaller hippocampi, fusiform and parahippocampal gyri) has been observed in Met allele carriers (Montag et al., 2009), which would lead one to expect a reduced emotional enhancement effect. However, increased emotion-related activity, despite the smaller volume, would suggest the opposite (Montag et al., 2008); similarly, a finding of significantly smaller hippocampi in Met carriers which were significantly *more* active during encoding of negative – but not neutral – words (with no difference in behavioral memory performance), suggests that the genotype is likely to effect emotional memory, potentially to a greater extent than neutral (Molendijk et al., 2012). While the neural evidence doesn’t present a clear picture of whether we would expect emotional memory performance would be increased or decreased in Met carriers compared to Val homozygotes, the results of two studies which found performance differences suggests that it would be lowered. Cathomas et al. (2010), for example, found significantly lower recall of positively valenced words in Met carriers compared to Val homozygotes; Keyan and Bryant (2017), also found differences in the recall of positive stimuli, in which Val homozygotes showed a post-exercise recall enhancement (interacting with cortisol), which was not observed in Met carriers.

As well as increasing the activation of amygdala and medial temporal regions, recognition of emotional stimuli also increases the old/new difference of the LPC component (Johansson et al., 2004; Inaba et al., 2005; Weymar et al., 2009; Schaefer et al., 2011; Xu et al., 2015; Meng et al., 2017). Some studies have reported modulations of the LPC by valence polarity and arousal magnitude, however, the directions are inconsistent (Xu et al., 2015; Meng et al., 2017).

We performed two separate EEG experiments on emotional memory in two independent populations genotyped for the BDNF Val66Met polymorphism. We analyzed the LPC during recognition memory of negative and neutral words (Experiment 1) and negative and positive words (Experiment 2). A directed forgetting design was used, in order to provide measures of both intentional and unintentional memory, as emotional enhancement effects tend to be largest when processing resources are constricted (Talmi, 2013). However, trial numbers were not sufficient to allow the analysis of memory for items which participants had been instructed to forget, and so our analysis was restricted to intentional memory only.

Based on the literature reviewed above, we predicted a significant difference between the ERP response to correctly rejected new words compared to successfully remembered words occurring at 500–800 ms and maximal in posterior compared to frontal regions, consistent with the topography of the LPC. We hypothesized that this LPC difference would be significantly reduced in Met carriers compared to Val homozygotes, and we tentatively hypothesized that this reduction would be largest in response to emotional (both positive and negative) stimuli.

## Experiment 1 Materials and Methods

The experimental protocols of both studies were approved by the local ethics committee at Goldsmiths University of London where testing took place. All procedures contributing to this work comply with the ethical standards of the relevant national and institutional committees on human experimentation and with the Helsinki Declaration of 1975, as revised in 2008.

### Participants

Subjects were recruited through an online university participation scheme and advertisements posted around Goldsmiths, University of London, and University of Westminster. Planned sample size was based on previous studies investigating the effect of the BDNF Val66Met polymorphism (e.g., Hariri et al., 2003; Montag et al., 2008; Keyan and Bryant, 2017); an *a priori* power analysis was not run. In Sample 1 (*N* = 56), three subjects had missing EEG data and five subjects had undetermined BDNF genotype; a further three participants were excluded due to insufficient numbers of epochs, and five more were excluded as they reported being diagnosed with a psychological illness, and a final three participants were excluded on the basis of low discrimination (following MacLeod and Donaldson, 2017). This left a final sample of 37 participants (30 female) aged 18–47 (24.89 ± 6.47) who were included in the analysis. Table 1 shows subjects’ demographics.

**TABLE 1:** Subject demographics as a function of each experiment and BDNF Val66Met genotype group.

	**Experiment 1 (*N* = 37)**		**Experiment 2 (*N* = 23)**	
	**Val homozygotes**	**Met carriers**	**Val homozygotes**	**Met carriers**
*N* (% of Sample)	25 (68%)	12 (32%)	11 (48%)	12 (52%)
Sex (male, female)	4, 21,	3, 9,	1, 10	4, 9
Age^1^ (years)	25.08 (7.54)	24.5 (3.53)	21.27 (3.38)	23.50 (3.21)
Nationality:				
United Kingdom	16	9	10	10
Europe	7	2	1	2
Other^2^	1	1		0
English first language^3^	–	–	11	9
BDI	8.24 (8.43)	5.08 (3.03	–	–
WBSI	47.04 (10.90)	44.42 (11.77)	–	–
OCI-R	14.16 (9.70)	13.58 (7.17)	29.55 (15.41)	9.13 (7.47)
STAI-T	50.56 (48.42)	59.58 (11.54)	–	–

*Met carrier group represents the combined total of heterozygous and homozygous carriers of the Met allele. ^1^Age undisclosed by 1 Val homozygote and 1 Met carrier from Sample 1; ^2^Nationality undisclosed by 1 Val homozygote from Sample 1; ^3^English first language not collected for sample 1. Key: BDNF Val66Met, single nucleotide polymorphism on the brain-derived neurotrophic factor gene leading to a valine to methionine amino acid substitution at codon 66; Val, valine; Met, methionine; BDI, beck depression inventory; OCI-R, obsessive-compulsive inventory – revised; STAI-T, spielberger state-trait anxiety inventory – trait subscale.*

### Measures

Subjects completed the Beck Depression Inventory II (Beck et al., 1961); the Obsessive-compulsive Inventory – Revised (Foa et al., 2002); the White Bear Suppression Inventory (Wegner and Zanakos, 1994), and the State-Trait Anxiety Inventory (Trait subscale; Spielberger et al., 1983). These measures were included as part of a larger study, and not of interest for the current analysis, however, between-group comparisons and correlations were included to rule out the effects of individual differences on our findings.

### Genotyping

Genomic DNA was extracted from buccal cells following the procedure described by Freeman et al. (2003). The BDNF Val66Met polymorphism was assayed using the rs6265 TaqMan SNP Genotyping Assay (Thermo Fisher Scientific, United States) and analyzed on a 7900HT Sequence Detection System (Thermo Fisher Scientific, United States).

### Stimuli

A total of 320 words were selected from the ANEW database (Bradley and Lang, 2017). Eighty neutral and 80 negative words were presented in the study phase, and a further 80 neutral and 80 negative words were presented in the test phase. Each study-phase list was sub-divided into instruction type as follows: to-be-remembered, TBR, and to-be-forgotten, TBF. Words from the two phases and instructions were matched for valence, arousal, frequency, and word length. Negative and neutral words differed significantly in valence, arousal, and frequency, but were matched for word length (Supplementary Table S1).

### Experimental Design and Procedure

Subjects were seated in a room with intercom connection to the experimenters. The session began with a 2 min resting state recording as part of a larger study.

Participants were then presented with instructions on a computer monitor 90 cm in front of them. These stated that they would be presented with words on screen which would each be followed by an instruction to remember (‘RRRRR’) or forget (‘FFFFF’) the word. They were informed that they would later be tested on their memory only for the words they had been instructed to remember.

The experiment began with a practice block (16 trials). In a typical trial: A white fixation cross began each trial (1000 ms), then turned red (500 ms) to indicate a study word was about to appear. The study word was then presented (500 ms), followed by a white fixation cross (1000 ms) which turned red (500 ms) before presentation of the instruction cue (500 ms). To minimize ocular artifacts, subjects were encouraged to blink when the fixation cross was white. The study phase then began, with a procedure identical to the practice, consisting of all the study list items, counterbalanced for instruction, beginning with four neutrally valenced ‘buffer’ items which were not included in later analysis. The study phase took approximately 10 min. After the study phase subjects performed a short Go/NoGo filler task, in which they were required to respond with a key press as quickly as possible when presented with a circle on screen (‘Go’, 80% of trials), and withhold their response when presented with a square (‘NoGo’, 20% of trials). This task took approximately 5 min, and was then followed by a short break (5 min) before the test phase.

The test phase presented old words from the TBR and TBF lists, intermixed with words from the new list, resulting in 320 trials, and lasting approximately 15 min. Subjects were asked to indicate if these words were new or old – regardless of whether they had been previously instructed to be remembered or forgotten. The test phase words were preceded by a white (1000 ms) and then red (500 ms) fixation cross, as in the study phase, and remained on screen until subjects submitted their response using a key-pad. E-Prime 2.0 software was used for stimulus presentation and response collection.

### Behavioral Data Analysis

The frequency of correct rejections (CR) and recognition (Hits) were calculated, as were sensitivity (Pr = pHit-pFA) and bias (Br = pFA/[1-Pr]). For sensitivity and bias, hit rates and false alarm (FA) rates were adjusted (Hit rate = Hit + 0.5/number of old stimuli + 1; FA rate = FA + 0.5/number of new stimuli + 1; see Snodgrass and Corwin, 1988), as in MacLeod and Donaldson (2017). CR, Hits, Pr, and Br were entered into separate ANOVAs with factors of *Valence* (negative, neutral), and *Genotype* (Val homozygotes, Mett carriers). Pearson’s correlations were run between questionnaire scores and behavioral performance for the combined sample within each experiment, and Bonferroni correction was applied as appropriate.

### EEG Recording

Sixty-four channel EEG was recorded by active scalp electrodes according to the 10–20 International system of electrode placement. Additional electrodes were placed above, below, and at the outer canthus of each eye, to record vertical and horizontal eye movements, respectively. The EEG signals were amplified by BioSemi Active Two^®^ amplifiers and filtered between 0.6 and 100 Hz. The sampling rate was 512 Hz.

### EEG Data Analysis

Data pre-processing was carried out using the EEGLAB v.13 (Delorme and Makeig, 2004) toolbox for MATLAB^®^. Test phase EEG data was re-referenced off-line to the average of the two earlobe electrodes, and filtered between 0.5 and 48 Hz (basic FIR filter).

Epochs were created with a duration of −500 – 1200 ms, time-locked to word onset and baseline corrected. Eye-movement related artifacts were removed using independent component analysis (ICA) method (1.69 ± 1.19 components per subject), bad channels were replaced using nearest neighbor interpolation (2.90 ± 2.23 channels per subject), and any epochs with remaining artifacts were rejected semi-automatically with a threshold of ±85 μV. There were no significant differences between genotype groups’ number of ICA components removed, channels interpolated, or epochs rejected (all *p* > 0.05). Following data cleaning there were sufficient (>10) epochs available for analysis of correctly identified but not incorrectly identified words. Two Met carriers and one Val homozygote were excluded from EEG analysis due to insufficient (≤10) numbers of epochs available for any condition.

Following Luck and Gaspelin (2017), care was taken to reduce the likelihood of bogus effects: in line with their guidance, only the ERP component relevant to the hypothesis was analyzed, and multiple comparisons were reduced by collapsing across hemispheres, and analyzing old-new contrasts rather than old and new ERP magnitudes separately. Furthermore, while effects or interactions testing specific hypotheses were tested at an alpha level of 0.05, those which did not test a specific hypothesis were tested according to a Bonferroni corrected alpha level – corrected for the seven contrasts within the ANOVA (0.5/7 = 0.007) to reduce familywise error.

Mean ERP amplitudes were calculated for frontal (AF3, AF7, F1, F3, F5, FC1, FC3, FC5, AF4, AF8, F2, F4, F6, FC2, FC4, and FC6) and posterior (C1, C3, CP1, CP3, CP5, P1, P3, P5, C2, C4, CP2, CP4, CP6, P2, P4, and P6) brain regions. Mean amplitudes for the 500–800 ms time window was extracted from these clusters, consistent with the typical timing of the LPC (e.g., Woodruff et al., 2006; Vilberg and Rugg, 2009; Weymar et al., 2009; Meng et al., 2017).

Mixed factorial ANOVA were run on old-new difference scores, with factors of *Valence* [neutral, negative] × *Caudality* [frontal, parietal] × *Genotype* [Val homozygote, Met carrier]. Spearman’s correlations were run between questionnaire scores and LPC magnitude, and Bonferroni correction was applied as appropriate.

For all experiments, we have reported all measures, conditions, data exclusions, and how we determined our sample sizes.

## Results

### Subject Characteristics

There were no significant differences between genotype groups of Sample 1 in age or sex distribution (*p* > 0.05). No significant differences in questionnaire responses were found between genotypes in Sample 1 (all *p* > 0.05).

### Behavioral Performance

Means and standard deviations for behavioral performance are shown in Table 2.

**TABLE 2:** Memory performance according to genotype group in Experiment 1.

	**Val homozygotes**	**Met carriers**
**Memory performance**		
Negative CR	60.00 ± 10.87	66.33 ± 8.86
Negative Hit	31.44 ± 5.12	27.17 ± 5.59
Negative PR	0.53 ± 0.16	0.50 ± 0.14
Negative Br	0.53 ± 0.23	0.36 ± 0.21
Neutral CR	67.08 ± 9.03	72.58 ± 5.96
Neutral Hit	28.72 ± 5.38	23.67 ± 6.36
Neutral PR	0.55 ± 0.15	0.49 ± 0.16
Neutral Br	0.37 ± 0.20	0.20 ± 0.14

*CR, correctly rejected new words; Hit, correctly recognized old words; PR, discriminability; BR, bias.*

#### Correct Rejection and Recognition Rates

**FIGURE 1:**
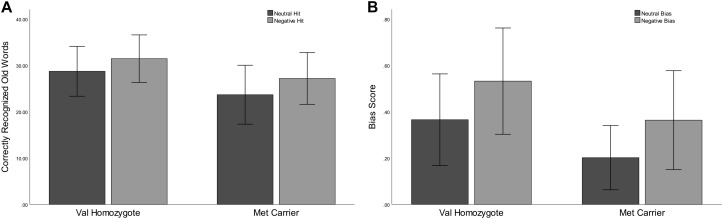
Experiment 1: Mean recognition accuracy as a function of valence for Val homozygotes (left within graph) and Met carriers (right within graph) **(A)**. Mean bias score as a function of valence for Val homozygotes (left within graph) and Met carriers (right within graph) **(B)**. Error bars show 1 standard deviation of the mean.

Correct rejection of new words (CR), and recognition of old words (Hits) were entered into two separate ANOVAs with factors of *Valence* and *Genotype*. These revealed significantly lower correct rejection of negative compared to neutral words [*F*(1,35) = 24.55, *p* < 0.001, $\eta {}_{p}^{2}$ = 0.41], and a strong trend toward higher correct rejection in Val homozygotes than Met carriers [*F*(1,35) = 3.96, *p* = 0.05, $\eta {}_{p}^{2}$ = 0.10], as shown in Figure 1. As predicted, the recognition rate for negative words was significantly greater than for neutral words [*F*(1,35) = 12.65, *p* = 0.001, $\eta {}_{p}^{2}$ = 0.27]. A significant main effect of *Genotype* showed significantly high recognition in Val homozygotes than Met carriers [*F*(1,35) = 7.35, *p* = 0.01, $\eta {}_{p}^{2}$ = 0.17]. No significant relationships were present between recognition/rejection accuracy and questionnaire scores, following Bonferroni correction.

#### Sensitivity and Bias

No significant effects of *Valence* or *Genotype* were observed on discrimination (all *p* > 0.05). However, a highly significant effect of *Valence* was found for Bias [*F*(1,35) = 29.11, *p* < 0.001, $\eta {}_{p}^{2}$ = 0.45], with significantly higher bias for negative words, and also a main effect of *Genotype* [*F*(1,35) = 6.56, *p* = 0.02, $\eta {}_{p}^{2}$ = 0.16], with significantly lower bias in Met carriers than Val homozygotes (see Figure 1).

### BDNF Val66Met Genotype Modulation of LPC During Emotional Word Recognition

**FIGURE 2:**
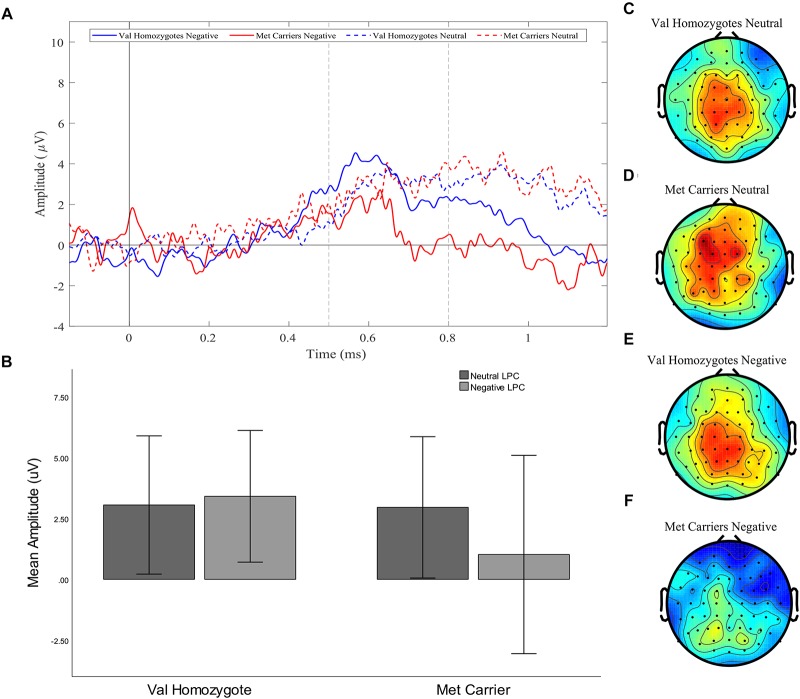
Experiment 1: Old-New difference waves for the Val homozygotes (blue) and Met carriers (red) in negative (solid lines) and neutral (dashed lines) conditions **(A)**. Mean (±1SD) LPC amplitude of Val homozygotes (left within graph) and Met carriers (right within graph) for the neutral (light gray) and negative (dark gray) conditions **(B)**. Topographic distribution of LPC effect between 500–800 ms in Val homozygotes neutral condition **(C)**, Met carriers neutral condition **(D)**, Val homozygotes negative condition **(E)**, and Met carriers negative condition **(F)**.

A significant main effect of *Caudality* confirmed the old-new difference to be largest in posterior regions, consistent with the topography of the LPC [*F*(1,35) = 10.455, *p* = 0.003, $\eta {}_{p}^{2}$ = 0.23]. *Genotype* interacted significantly with *Valence* and *Caudality* [*F*(1,35) = 7.78, = 0.009, $\eta {}_{p}^{2}$ = 0.18], but not with *Caudality* alone [*F*(1,35) = 2.04, *p* = 0.16, $\eta {}_{p}^{2}$ = 0.06]. Independent samples *t*-tests were run on negative and neutral old-new differences in posterior regions, showing a significantly larger old-new difference in Val homozygotes than Met carriers in the negative condition [*t*(2,35) = 2.13, *p* = 0.04] but not in the neutral condition [*t*(2,35) = 0.10, *p* = 0.92], as illustrated in Figure 2.

No effects or interactions which had not been hypothesized survived correction for multiple comparisons (see Table 3).

**TABLE 3:** Experiment 1: Results of mixed factorial ANOVA of old-new difference scores with factors of Valence (2) × Caudality (2) × Group (2).

**Contrast**	***F*(*p*)**	**$\eta {}_{p}^{2}$**	**Direction (significant main effects only)**
**Hypothesized effects and interactions (α = 0.05)**			
Caudality	10.46 (0.003)^∗∗^	0.23	Posterior > Frontal
Caudality × Genotype	2.04 (0.16)	0.06	–
Valence × Caudality × Genotype	7.78 (0.009)^∗^	0.18	–
**Non-hypothesized effects and interactions, applying Bonferroni correction according to 7 comparisons (alpha of 0.5/7 = 0.007)**			
Valence	3.20 (0.082)	0.08	–
Valence × Genotype	5.48 (0.025)	0.14	–
Valence × Caudality	7.86 (0.008)	0.18	–
Genotype	1.31 (0.26)	0.04	–

*Effects and interactions specifically addressing the hypothesis are tested according to an alpha level of *p* < 0.05; Effects and interactions not addressing a hypothesis are tested according to a Bonferroni corrected alpha level of *p* < 0.007. ^∗^*p* < 0.05, ^∗∗^*p* < 0.005; degrees of freedom = 1,35.*

## Experiment 1 Summary

Behaviorally, our results showed a typical emotional enhancement effect, with negative words recognized significantly more frequently than neutral words. Superior memory performance was observed in Val homozygotes than Met carriers, but there was no interaction with valence. The findings of our LPC analysis were in partial agreement with our hypothesis, as we saw an interaction between *Genotype* and *Valence*, showing Met carriers were associated with a reduced LPC, but only in the negative condition.

In order to replicate and extend the findings of Experiment 1, a second experiment was run, with an independent sample, to assess whether Met carriers would have a reduced LPC in a positive condition as well as the negative. In this way, we sought to delineate whether the genotype differences were due to emotional arousal or valence. Based on previous findings of reduced memory for positively valenced stimuli (Cathomas et al., 2010; Keyan and Bryant, 2017), we predicted that there would be no interaction of *Genotype* with *Valence* when using negative and positive conditions, reflecting an effect of emotional arousal independent of valence polarity.

## Experiment 2 Materials and Methods

### Participants

Subjects were recruited through an online university participation scheme and advertisements posted around Goldsmiths, University of London, and University of Westminster. Planned sample size was based on previous studies investigating the effect of the BDNF Val66Met polymorphism (e.g., Hariri et al., 2003; Montag et al., 2008; Keyan and Bryant, 2017); an *a priori* power analysis was not run. In Sample 2 (*N* = 31), two subjects had missing EEG data and one subject’s BDNF genotype was undetermined; twenty-eight subjects (4 male, 24 female, aged 18–33 years) were included in the analysis. Table 1 shows subjects’ demographics.

As in Experiment 1, participants with discrimination scores lower than 0.2 were excluded from analysis. Following exclusions, there were 23 participants in the final Experiment 2 sample (19 females), aged 18–28 (22.43 ± 3.41). Participant demographics according to genotype can be found in Table 1.

### Measures

Subjects completed the Obsessive-compulsive Inventory – Revised (Foa et al., 2002); As in Experiment 1, these measures are not of interest for the current analysis, however, between-group comparisons and correlations were included to rule out the effects of individual differences on our findings (Table 1).

### Genotyping

Genomic DNA was extracted and genotyped as in Experiment 1.

### Stimuli

Neutral words from Experiment 1 were replaced with 160 positive words from the ANEW database (40 study TBR, 40 study TBF, 80 test). Words from the two phases and instruction types were matched for valence, arousal, frequency, and word length. Negative and positive words were matched for arousal, frequency, and word length (Supplementary Table S2).

### Experimental Design

Experiments 2 was identical to Experiment 1 except for valence conditions.

### Behavioral Data Analysis

Behavioral data analysis was carried out as in Experiment 1.

### EEG Recording

Sixty-four channel EEG was recorded as in Experiment 1.

### EEG Data Analysis

Data pre-processing was carried out as in Experiment 1. Ocular artifacts were removed using ICA (1.36 ± 0.68 components removed per participant), and bad channels were replaced using nearest neighbor interpolation (3.0 ± 2.28 channels per participant), and any epochs with remaining artifacts were rejected semi-automatically with a threshold of ±85 μV, as in Experiment 1. There were no significant differences between genotype groups’ number of ICA components removed, channels interpolated, or epochs rejected (all *p* > 0.05).

Mean ERP amplitudes were calculated as above. Mixed factorial ANOVA were run on old-new difference scores, with factors of *Valence* [negative, positive] × *Caudality* [frontal, posterior] × *Genotype* [Val homozygote, Met carrier]. Spearman’s correlations were run between questionnaire scores and LPC magnitude, and Bonferroni correction was applied as appropriate.

## Results

### Subject Characteristics

In Sample 2 there was no significant difference between the genotype groups’ age (*p* > 0.05), and although there was only one male Val homozygote, the association between *Genotype* and *sex* did not reach significance (χ*^2^* = 1.98, *p* > 0.05). However, Val homozygotes did score significantly higher than Met carriers on the OCI-R (*t* = 3.44, *p* < 0.01).

### Behavioral Performance

Means and standard deviations of behavioral performance can be found in Table 4.

**TABLE 4:** Memory performance according to genotype group in Experiment 2.

	**Val homozygotes**	**Met carriers**
**Memory Performance**		
Negative CR	65.09 ± 10.20	61.67 ± 10.31
Negative Hit	28.64 ± 5.35	28.58 ± 3.53
Negative PR	0.52 ± 0.11	0.48 ± 0.10
Negative Br	0.38 ± 0.24	0.43 ± 0.18
Positive CR	69.18 ± 7.10	67.42 ± 6.96
Positive Hit	25.36 ± 6.41	28.42 ± 3.40
Positive PR	0.49 ± 0.14	0.54 ± 0.12
Positive Br	0.29 ± 0.20	0.35 ± 0.12

*CR, correctly rejected new words; Hit, correctly recognized old words; PR, discriminability; BR, bias.*

#### Correct Rejection and Recognition Rates

**FIGURE 3:**
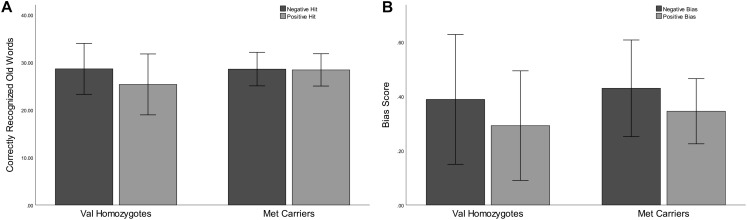
Experiment 2: Mean recognition accuracy as a function of valence for Val homozygotes (left within graph) and Met carriers (right within graph) **(A)**. Mean bias score as a function of valence for Val homozygotes (left within graph) and Met carriers (right within graph) **(B)**. Error bars show 1 standard deviation of the mean.

Lower correct rejection of negative compared to positive words was seen in Experiment 2 [*F*(1,21) = 18.79, *p* < 0.001, $\eta {}_{p}^{2}$ = 0.47], but no genotype difference was present [*F*(1,21) = 0.5, *p* = 0.47, $\eta {}_{p}^{2}$ = 0.03]. Correct recognition was superior for negative compared to positive words [*F*(1,21) = 5.168, *p* = 0.03, $\eta {}_{p}^{2}$ = 0.20]. No main effect of *Genotype* was present [*F*(1,21) = 0.66, *p* = 0.43, $\eta {}_{p}^{2}$ = 0.03] as shown in Figure 3. No significant relationships were present between recognition/rejection accuracy and questionnaire scores, following Bonferroni correction.

#### Sensitivity and Bias

No significant effects of *Valence* or *Genotype* were observed for discrimination (all *p* > 0.05). Once again, there was a highly significant effect of *Valence* on bias [*F*(1,21) = 20.96, *p* < 0.001, $\eta {}_{p}^{2}$ = 0.50], as there was higher bias in the negative than the positive condition. No *Genotype* effects were present (all *p* > 0.05; see Figure 3).

### BDNF Val66Met Genotype Modulation of LPC During Emotional Word Recognition

**FIGURE 4:**
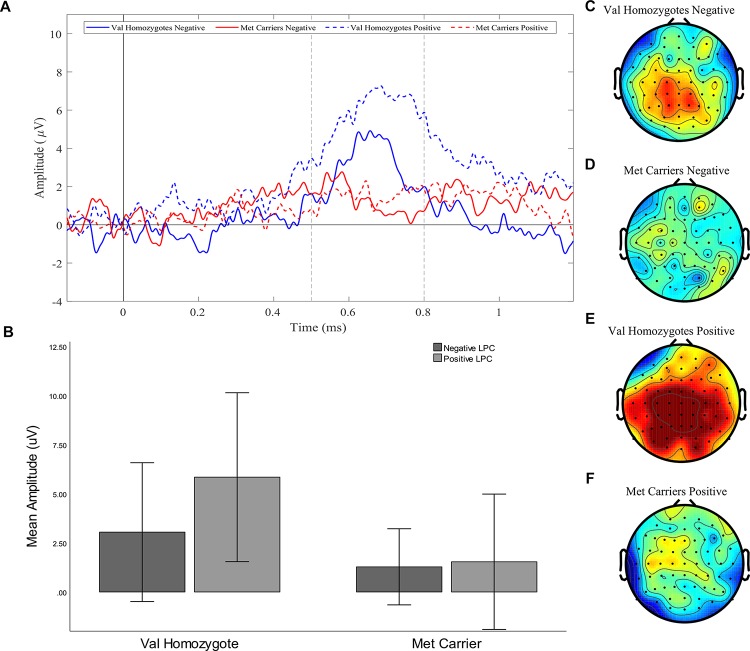
Experiment 1: Old-New difference waves for the Val homozygotes (blue) and Met carriers (red) in negative (solid lines) and positive (dashed lines) conditions **(A)**. Mean (±1SD) LPC amplitude of Val homozygotes (left within graph) and Met carriers (right within graph) for the positive (light gray) and negative (dark gray) conditions **(B)**. Topographic distribution of LPC effect between 500–800 ms in Val homozygotes negative condition **(C)**, Met carriers negative condition **(D)**, Val homozygotes positive condition **(E)**, and Met carriers positive condition **(F)**.

A significant main effect of *Caudality* once again confirmed that the old-new difference was largest in posterior regions [*F*(1,21) = 5.39, *p* = 0.03, $\eta {}_{p}^{2}$ = 0.20, and the expected interaction of *Caudality* and *Genotype* was also observed *F*(1,22) = 4.51, *p* = 0.046, $\eta {}_{p}^{2}$ = 0.18]. Within posterior regions, the main effect of *Genotype* showed a significantly greater old-new effect was seen in Val homozygotes than Met carriers [*F*(1,21) = 6.27, *p* = 0.02, $\eta {}_{p}^{2}$ = 0.23], as illustrated in Figure 4. No effects or interactions which had not been hypothesized survived correction for multiple comparisons (see Table 5). In order to ensure that the unequal gender distribution across genotype groups was not having an effect on our results, the analysis of posterior electrodes was re-run with *sex* as a covariate. No significant effects of sex were observed (all *p* > 05), and the main effect of *Genotype* remained significant [*F*(1,20) = 9.33, *p* = 0.01, $\eta {}_{p}^{2}$ = 0.32].

**TABLE 5:** Experiment 2: Results of mixed factorial ANOVA of old-new difference scores with factors of.

**Valence (2) × Caudality (2) × Genotype (2)**			

**Contrast**	***F*(*p*)**	**$\eta {}_{p}^{2}$**	**Direction (significant main effects only)**
**Hypothesized effects and interactions (α = 0.05)**			
Caudality	5.39 (0.03)	0.20	Posterior > Frontal
Caudality × Genotype	4.51 (0.046)	0.18	
**Non-hypothesized effects and interactions, applying Bonferroni correction according to seven comparisons (alpha of 0.5/7 = 0.007)**			
Valence	4.65 (0.043)	0.18	
Valence × Genotype	3.35 (0.082)	0.14	
Valence × Caudality	0.77 (0.388)	0.04	
Valence × Caudality × Genotype	0.41 (0.53)	0.02	
Genotype	4.49 (0.046)	0.18	

**Valence (2) × Group (2), within posterior regions**			
**Hypothesized effects and interactions (α = 0.05)**			
Genotype	6.27 (0.021)	0.23	Val homozygotes > Met carriers
**Non-hypothesized effects and interactions, applying Bonferroni correction according to seven comparisons (alpha of 0.5/3 = 0.007)**			
Valence	4.42 (0.048)	0.17	
Valence × Genotype	3.06 (0.10)	0.13	

*Effects and interactions specifically addressing the hypothesis are tested according to an alpha level of *p* < 0.05; Effects and interactions not addressing a hypothesis are tested according to a Bonferroni corrected alpha level. ^∗^*p* < 0.05, ^∗∗^*p* < 0.005; degrees of freedom = 1,21.*

## Discussion

Our two electrophysiological experiments showed a consistent modulation of the LPC by BDNF Val66Met genotype and emotion. Specifically, they showed a significant reduction of the LPC amplitude in Met carriers compared to Val homozygotes when recognizing both negative and positive, but not neutral, words. As this effect was seen for both negative and positive stimuli, it is likely that arousal rather than valence mediated the effect. Although performance differences were seen between genotype groups in Experiment 1, we believe it is unlikely that these are responsible for the observed effect, for two reasons: (i) the performance difference was present for both valence conditions, whereas the ERP effect was only seen in the negative condition; (ii) no performance differences were seen in Experiment 2, where the effect was replicated.

Our findings are in agreement with the results of Hariri et al. (2003), who also found that while Met carriers had reduced recognition-related hippocampal activity than Val homozygotes, it was not related to their performance on the memory task; only their reduced encoding activity predicted their memory deficit. A wide body of evidence links the magnitude of the LPC to the strength or depth of episodic recollection. For example, the LPC is consistently found when participants give “remember” rather than “know” responses in the R/K paradigm (Smith, 1993; Rugg et al., 1998), and when individuals make correct source judgments (Wilding and Rugg, 1996) especially when they are made with a high level of confidence (Woroch and Gonsalves, 2010; Addante et al., 2012). The LPC is widely agreed to index conscious episodic recollection (Woodruff et al., 2006; Yu and Rugg, 2010), particularly the amount of episodic detail retrieved (Wilding, 2000; Vilberg et al., 2006; Vilberg and Rugg, 2009). A reduced LPC can also be a marker of recollection avoidance; Bergström et al. (2008), for example, found that voluntarily avoiding recollection of a paired associate word would significantly reduce, or even reverse, the LPC.

Episodic recollection is not necessary for successful recognition, however, it is associated with stronger and more confident memories (Woodruff et al., 2006). Studies attempting to use the LPC to classify good or poor memory performance have had mixed results (Curran and Cleary, 2003; Schiltz et al., 2006; MacLeod and Donaldson, 2017). Curran and Cleary (2003) found difference in the LPC between participants with good and poor recollection discriminability. However, in a recent, large-scale (*N* = 122) study specifically aimed at identifying whether the LPC was related to between participant measures of recollection, MacLeod and Donaldson (2017) found no difference between good and poor performers, and no correlation with discrimination accuracy on a simple old/new task, or a source memory task with remember/know/guess classifications.

Taken together, this strongly suggests a difference between BDNF Val homozygotes and Met carriers in the conscious experience of recollection, especially regarding emotional material. On the basis of previous studies examining the LPC, we suggest that Met carriers’ recollection of emotional material is likely to be less detailed and less confident than one would see in Val homozygotes.

While it might seem counterintuitive to suggest that individuals carrying the BDNF Met allele would have reduced recollection of emotional stimuli, as this genotype is associated with post-traumatic stress disorder (PTSD), it is not without precedent; an fMRI study of PTSD flashbacks has shown that neurally the are more similar to a strong sense of familiarity than to recollection (Whalley et al., 2013). Reduced recollection of emotional episodic detail may also provide an explanation for the poor response to exposure therapy seen in PTSD patients carrying the Met allele, compared to homozygous Val carriers (Felmingham et al., 2013). As the authors suggest, it is likely that this reflects BDNF-related abnormalities in prefrontal-amygdalar networks related to fear extinction; however, it may also be the case that reduced recollection impedes the reconsolidation mechanisms underlying exposure therapy, as this relies heavily on reactivation of the traumatic memory (see Beckers and Kindt, 2017).

Reduced recollection is strongly related to overgeneral memory (OGM), positively correlating in both healthy controls and schizophrenic subjects (Danion et al., 2005; Neumann et al., 2007). Our results may, therefore, indicate a predisposition toward OGM in Met carriers, at least for emotional stimuli. This is supported by evidence that the hippocampus, which has been consistently implicated in recollection rather than familiarity (Montaldi and Mayes, 2010), is a vital constituent of LPC generation (Düzel et al., 2001; Vilberg and Rugg, 2009; Addante et al., 2012; Hoppstädter et al., 2015), It has recently been theorized that OGM in depression is the result of stress-induced suppression of neurogenesis in the dentate gyrus of the hippocampus, leading to deficient “pattern separation” and hence difficulty creating unique memories for similar events (Dillon and Pizzagalli, 2018). Crucially, this process of pattern separation, which occurs during the encoding/consolidation stage of memory in the dentate gyrus, is dependent on BDNF (Bekinschtein et al., 2014).

Although we predicted that Met carriers’ LPC amplitude would be reduced most prominently in the emotional conditions, we also expected a reduction in the neutral LPC (albeit smaller), due to the hippocampus’s crucial role in producing the component. However, Experiment 1 showed no differences between the Met carriers and Val homozygotes’ LPC in the neutral condition. As previous studies investigating the role of BDNF Val66Met in memory for emotional and neutral stimuli combined also tend to find group differences only in the emotional but not the neutral condition (e.g., Montag et al., 2008; Cathomas et al., 2010; Molendijk et al., 2012; Keyan and Bryant, 2017), our finding is not entirely unexpected, but is interesting. The necessity of the emotional aspect for a group difference to be observed suggests that it is due to abnormalities involving amygdalar circuitry rather than the hippocampus alone.

The current studies are not without limitations. The primary limitation is the statistical power. Both of our experiments – and especially Experiment 2 – have very low sample sizes, and so are certainly underpowered. While our planned sample sizes were based on previous studies investigating the effect of the BDNF Val66Met polymorphism on memory (e.g., Hariri et al., 2003; Montag et al., 2008; Keyan and Bryant, 2017), the use of an a prior power analysis to determine adequate sample size would be far more preferable, and should be used in future. However, the use of a partial replication, showing the reduced LPC to emotional stimuli in two separate experiments with independent samples, adds weight to our findings; as replication can be seen as the most important approach to acquiring valid results (see Luck and Gaspelin, 2017), we consider our findings to be worthy of reporting, but in need of further replication.

Regarding sampling, a further limitation lies in the gender distribution of Experiment 2, in which there are no Val homozygotes. Gender differences are often reported in studies of emotion processing, including an enhanced amygdala response to affective stimuli in males (see Kret and De Gelder, 2012). There is limited evidence regarding the potential effects of gender on the LPC component, however, some data suggests differences in hemispheric lateralization – with females showing a more right-lateralized, and males a more left-lateralized, topography during emotional recognition (Lavoie et al., 2016). The inclusion of sex as a covariate in Experiment 2 did not diminish the effect of BDNF genotype on the LPC – and in fact increased the effect size – suggesting that our results were not due to gender differences. However, as the samples of both our experiments are primarily female, future studies should aim to identify whether the BDNF Met genotype has a similar effect in male-only samples.

A further limitation, which prevents us from stating unequivocally that our results reflect reduced emotional episodic recollection in Met carriers, is that subjective recollection and familiarity judgments were not collected. While the abundance of evidence linking the LPC component to conscious recollection leads us to be confident in our hypothesis, future studies incorporating subjective judgments are necessary. We suggest the incorporation of both ‘remember’, ‘know’ and ‘guess’ responses, and confidence ratings, into future replications; on the basis of our findings and previous LPC studies we predict a reduced LPC amplitude in Met carriers to be accompanied by lower confidence estimates and a lower frequency of ‘remember’ judgments, consistent with lower recollection.

Also, we were unable to analyze ERPs elicited by words which participants had initially been instructed to forget. Unfortunately, in the current study, there were not a sufficient number of epochs for this condition to be included; trial numbers were simply not sufficient for analysis. As a reduction of the LPC has been linked to avoidance of recollection (Bergström et al., 2008), and emotional memory differences tend to be amplified when processing resources are limited (Talmi, 2013) we might expect group differences to be larger in this condition, so future studies may benefit from replicating with only negatively valenced words in order to maximize the number of available epochs.

Although the total duration of the experiment was reasonably short (not exceeding 40 min, inclusive of breaks), a large number of stimuli were presented, and it is therefore possible that attention waned toward the end. However, the number is standard for experiments of this kind (e.g., Gallant and Dyson, 2016), and the removal of participants with especially low discrimination scores should have minimized the effect of fatigue-induced guessing; furthermore, there is no reason to believe that reduced attention would effect one group more than the other, so systematic error is unlikely.

In summary, our results have shown that emotional recognition in BDNF Val66Met carriers is accompanied by a neural signature characteristic of reduced conscious episodic recollection. On this basis, we suggest that the role of this genotype in explicit memory biases warrants further attention.

## Data Availability

All data for this study is now available at: https://osf.io/sk987/.

## Ethics Statement

This study was carried out in accordance with the recommendations of the local ethics committee at Goldsmiths, University of London with written informed consent from all subjects. All subjects gave written informed consent in accordance with the Declaration of Helsinki. The protocol was approved by the local ethics committee at Goldsmiths.

## Author Contributions

RJ and JB devised the project, and wrote the manuscript with contributions from GC. RJ collected and analyzed the EEG data. GC carried out the genotyping.

## Conflict of Interest Statement

The authors declare that the research was conducted in the absence of any commercial or financial relationships that could be construed as a potential conflict of interest.
