# Supplementary material for: Brain-Derived Neurotrophic Factor Val66Met Polymorphism Is Associated With a Reduced ERP Component Indexing Emotional Recollection
Source: Front Psychol. 2019 Aug 21;10:1922. doi: 10.3389/fpsyg.2019.01922 (PMC6712090; doi:10.3389/fpsyg.2019.01922)
Supplement: Supplementary file 1 [file Table_1.DOCX]

**Brain-derived neurotrophic factor Val66Met polymorphism is associated with a reduced ERP component indexing emotional recollection**

Supplementary Information

| **Table S1:** Study and test stimuli matched for valence, arousal, word length and Francis and Kučera frequency, from the ANEW database. Table shows means and standard deviations of stimulus lists, and paired t-test results of comparisons between lists. | | | |
| --- | --- | --- | --- |
|  | Study Phase | | |
|  | Negative | Neutral | Negative vs. Neutral *t* (*p*) |
| Valence | 2.27 (.44) | 5.35 (.34) | 60.51 (< .001) |
| Arousal | 5.79 (.89) | 4.08 (.60) | 14.54 (< .001) |
| Word Length | 5.88 (1.30) | 5.79 (1.46) | .411 (.68) |
| Frequency | 24.99 (39.58) | 54.71 (60.70) | 3.52 (.001) |
|  | Test Phase | | |
|  | Negative | Neutral | Negative vs. Neutral *t* (*p*) |
| Valence | 2.34 (.42) | 5.35 (.36) | 68.99 (< .001) |
| Arousal | 5.82 (.92) | 4.10 (.71) | 12.97 (< .001) |
| Word Length | 5.90 (1.43) | 5.78 (1.43) | .57 (.57) |
| Frequency | 25.09 (61.26) | 55.83 (80.50) | 3.14 (.002) |
|  | Study Phase vs. Test Phase | | |
|  |  |  | Negative Study vs. Negative Test *t* (*p*) |
| Valence | - | - | 1.70 (.09) |
| Arousal | - | - | .10 (.92) |
| Word Length | - | - | .19 (.85) |
| Frequency | - | - | .17 (.87) |
|  |  |  | Neutral Study vs. Neutral Test *t* (*p*) |
| Valence | - | - | .11 (.92) |
| Arousal | - | - | .05 (.96) |
| Word Length | - | - | .08 (.94) |
| Frequency |  |  | .29 (.78) |

^ANEW, Affective Norms for English Words^

| **Table S2:** Study and test stimuli matched for valence, arousal, word length and Francis and Kučera frequency, from the ANEW database. Table shows means and standard deviations of stimulus lists, and paired t-test results of comparisons between lists. | | | |
| --- | --- | --- | --- |
|  | Study Phase | | |
|  | Negative  Mean (s.d) | Positive  Mean (s.d) | Negative vs. Positive *t* (*p*) |
| Valence | 2.57 (.63) | 7.11 (.65) | 45. 76 (<.001) |
| Arousal | 5.49 (1.08) | 5.35 (.91) | .86 (.39) |
| Word Length | 5.71 (.97) | 5.68 (1.06) | .21 (.83) |
| Frequency | 16.67 (22.17) | 16.55 (11.37) | .06 (.95) |
|  | Test Phase | | |
|  | Negative  Mean (s.d) | Positive  Mean (s.d) | Negative vs. Positive *t* (*p*) |
| Valence | 2.56 (.59) | 7.13 (.62) | 47.84 (<.001) |
| Arousal | 5.60 (0.91) | 5.33 (1.05) | 1.80 (.08) |
| Word Length | 5.69 (1.10) | 5.65 (1.04) | .24 (.81) |
| Frequency | 16.53 (26.40) | 16.31(15.16) | .11 (.91) |
|  | Study Phase vs. Test Phase | | |
|  |  |  | Negative Study vs. Negative Test *t* (*p*) |
| Valence | - | - | .44 (.66) |
| Arousal | - | - | .64 (.52) |
| Word Length | - | - | .15 (.88) |
| Frequency | - | - | .02 (.98) |
|  |  |  | Positive Study vs. Positive Test *t* (*p*) |
| Valence | - | - | .21 (.84) |
| Arousal | - | - | .15 (.89) |
| Word Length | - | - | .16 (.87) |
| Frequency |  |  | .51 (.62) |
| ^ANEW, Affective Norms for English Words^ | | | |
